# The Influence of the Reduction in Clay Sediments in the Level of Metals Bioavailability—An Investigation in Liujiang River Basin after Wet Season

**DOI:** 10.3390/ijerph192214988

**Published:** 2022-11-14

**Authors:** Xiongyi Miao, Jianping Liang, Yupei Hao, Wanjun Zhang, Yincai Xie, Hucai Zhang

**Affiliations:** 1 School of Geography and Environmental Science, Guizhou Normal University, Guiyang 550001, China; 2 School of Karst Science, Guizhou Normal University, Guiyang 550001, China; 3 Key Laboratory of Karst Dynamics, MNR&GZAR, Institute of Karst Geology, CAGS, Guilin 541004, China; 4 Guilin Meteorological Bureau of Guangxi, Guilin 541000, China; 5 Institute for Ecological Research and Pollution Control of Plateau Lakes, School of Ecology and Environmental Science, Yunnan University, Kunming 650500, China

**Keywords:** metals bioavailability, sediments, metals transformation, seasonal variation

## Abstract

The seasonal elevation of metals’ bioavailability can aggravate the threat of metal contamination in the aquatic environment. Nevertheless, their regulations have rarely been studied, particularly the connections between metals’ transformation and environmental variations. Therefore, the catchment area of Liujiang River was taken as an example in this study, their seasonal variations in metals’ bioavailability in sediments, especially during the wet season, was investigated to recover the processes associated with metals’ speciations and multiple environmental factors. The results revealed that the concentration of metals in sediments were high overall in the wet season, but low in the dry season. The significantly reduced ratio of metals in non-residual forms was largely related to the overall reduction in metals in oxidizable and reducible forms after the wet season. However, the elevated BI indexes of most metals suggested their increased bioavailability in the dry season, which should be closely related to their corresponding elevations in carbonate-bound and exchangeable forms after the wet season. The variations in metals’ bioavailability were primarily related to their predominance of exchangeable and carbonate-bound form. The higher correlation coefficients suggested the destabilization of the oxidizable form should be treated as a critical approach to the impact of metals’ bioavailability after the wet season. In view of that, sediments’ coarsening would pose the impacts on the destabilization of exogenous metals in sediments, the reduction in clay sediments should be responsible for the elevation of metals bioavailability after the wet season. Therefore, the monitoring of metals’ bioavailability in sediments should be indispensable to prevent metal contamination from enlarging the scope of their threat to the aquatic environment of the river, especially after the wet season.

## 1. Introduction

Rivers are the critical channels for the water cycle on earth, which plays an important role in the global transmission and transportation of materials and energy, but more and more sewage emission is being discharged into rivers with the development of industry. The emission of sewage significantly aggravates the aggregation of various contaminants in sediments, particularly heavy metals, which strongly degrade the ecological functions of the river [1]. The toxicological effects of metal contamination were formerly confirmed to be closely related to their bioconversion, so that the bioavailability of metals, instead of their total contents, were formerly proposed to determine the ecological risk of metal pollution in the sediments of rivers [2,3,4]. Despite metals’ bioavailability being widely used for assessing metal contamination in rivers with different functions, such as urban rivers [5], underground rivers [6] and artificial channels [7], etc., the seasonal variations in metals’ bioavailability in sediments were rarely reported, which was mainly because of the general concept that the sedimentary system was considered to be of sufficiently high stability to resist the impacts of environmental fluctuations on metals’ bioavailability [8]. However, the seasonal variations in metal bioaccumulation were successively found to be significant in various aquatic biotas all over the world, such as fishes [9], algae [10], and planktons [11], etc., which suggested the bioconversion of metals in rivers was variable, instead of invariable. As the predominant source of metals in river, sediments may be hard to keep from these modifications to metals’ bioconversion in rivers. To gauge the seasonal variations in metals’ bioconversion from sediments and to improve seasonal prevention and control of metal contamination in rivers, it is indispensable to strengthen the seasonal monitoring of metal bioavailability in sediments.

Metals can be preserved in a variety of chemical forms in sediments, which are closely related to the active substances they combine with, e.g., organic matter, sulfide, carbonate, and Fe/Mn oxy-hydroxide [2,12,13]. Multiple analysis methods have been proposed to simplify the existing forms of metals [3,12,14,15], the most widely used of which was proposed by Tessier and included exchangeable, carbonate-bound, reducible, oxidizable, and residual fractions [16]. Given the higher bioavailability of metals in exchangeable fractions and carbonate-bound fractions [4], they were commonly treated as the index of metals’ bioavailability to assess the influence of metal pollution on the environment [3]. In order to mitigate the negative impact of metal contamination, the chemical agents [17], hydrophytes [18], and microbes [19] were, respectively, proposed to trigger the transformation of exogenous metals from the exchangeable fractions and carbonate-bound fractions to reducible and oxidizable fractions, which aims to decrease metals’ bioavailability in sediments. However, the metals’ transformation that related to the decrease in metals’ bioavailability in sediments was not their entire process of metals’ transformation. Although metals in reducible and oxidizable fractions are considered to be relatively non-bioavailable [1,20], they could still reverse-transform to exchangeable and carbonate-bound forms, particularly with the influence of environmental variations. Even the inert residual form of metals could still undergo the destabilizing transformation [21,22], which is largely mediated by microorganisms [23], to exert their impacts on metals’ bioavailability. Therefore, the reverse transformations of metals in sediments were commonly treated as the great threat to the environmental security of rivers [24]. However, given that the reverse transformations of metals that are relevant to their destabilization were rarely incorporated into previous studies, their corresponding regulations and environmental effects still remained unclear. Facing the diversification of the reverse transformations of metals, it is an urgent need to determine the predominant pathway of metals reverse-transformation that dominate the variations in metals’ bioavailability, which is of great value in improving the understanding of the regulations that result from metals’ transformation on their bioavailability.

The watershed of Liujiang River is a typical subtropical karst catchment in southwest China, located in Liuzhou city, which is a regional economic center and the largest industrial city in Guangxi Province. More than 350 million tons of wastewaters from the extensive industrial operations were discharged into the watershed of Liujiang River annually, which eventually elevated metals’ contamination in the Liujiang River and its tributary, the Luoqingjiang River [25]. In recent years, the strict restriction on sewage discharge has substantially improved the water quality in these waterways, but exogenous metals still largely remained in the sediments, most of which were primarily preserved as non-residual forms [26]. It is well-known that metals in non-residual forms were treated as the critical parts to support their bioconversion [12], so that the contamination of metals in sediments gradually elevated metals’ bioaccumulation in the fish of the Liujiang River Basin [1]. Given concerns about the escalation in the bioconversion rate of metals in monsoon season, the investigation of metals’ bioavailability in sediments were carried out during the monsoon [2]. However, with the existence of the totally different environmental background in the dry season [27], the regulation of metals’ bioavailability make it difficult to go through the same processes of environmental variations as in the wet season, particularly the predominant approaches of metal transformation that dominate the variation in metals’ bioavailability may be also changed with the alternation of the seasons. Therefore, it is inadequate to determine the role of environmental fluctuations in regulating metals’ bioavailability by only relying on a one time survey in the monsoon. Currently, the seasonal variations in metals were successively reported to be significant either in the water and or the fish of the Liujiang River Basin [9,28], which suggested the management of metal contamination in the Liujiang River Basin may need to focus on their seasonal variations. Therefore, the seasonal environmental fluctuations should be incorporated into the simultaneous variations in the transformation and bioavailability of metals in sediments, which would not only benefit in determining the critical environmental factors that dominated the seasonal variations in metals’ bioavailability, but also could help to comprehensively elucidate the regulation of metals’ bioavailability in sediments. For this purpose, this study intended to (1) investigate the seasonal variations in metals’ speciations and their bioavailability in sediments; (2) determine the regulation of metals’ speciations on their bioavailability in different seasons; and (3) to ascertain the impacts of environmental fluctuations on metals’ bioavailability in different seasons, which condemned to have a great impact on the seasonal management of metal contamination in the Rivers.

## 2. Materials and Methods

### 2.1. The Description of the Study Area and Field Sampling

Liujiang River and Luoqingjiang River are the most important surface runoffs of Liujiang River Basin, the whole area of which is 58,398 ${\mathrm{km}}^{2}$. Among them, Liujiang River is the largest river in Liuzhou City with a total length of 272 km, which flows through the most functional areas of Liuzhou City from the confluence of Duliujiang River and Longjiang in Fengshan Town, while, with the exception of some industrial parks and part industrial areas in the east suburban areas of Liuzhou City, the watershed of Luoqingjiang River is mostly centralized in the rural areas without any operation of industries [25]. The intact karstic groundwater system is generated in the wide distribution of carbonate and karst landforms in Liuzhou River Basin, so that the alkaline groundwater from the karstic groundwater system is continuously recharged to Liujiang River and Luoqingjiang River, which results in the water being alkaline in these rivers all year round [28]. The gradients of Liujiang River and Luoqingjiang River are 1.68% and 0.55%, respectively. Due to the lush vegetation, the suspended particles in surface water are commonly less than 0.12 kg/m^3^ in the entire catchment of Liujiang River, their elevation was only found to be significantly higher in the wet season. The annual runoff amount of Liujiang River and Luoqingjiang River is 41 and 7.99 billion m^3^, respectively, more than 70% of which is concentrated in the wet season. The annual depth of the surface runoff in Liujiang River and Luoqingjiang River are 9 m and 5 m, respectively, the seasonal variations of which are over three times. All of this suggests that Liujiang River and Luoqingjiang River are heavily impacted by seasonal alternation.

**Figure 1:**
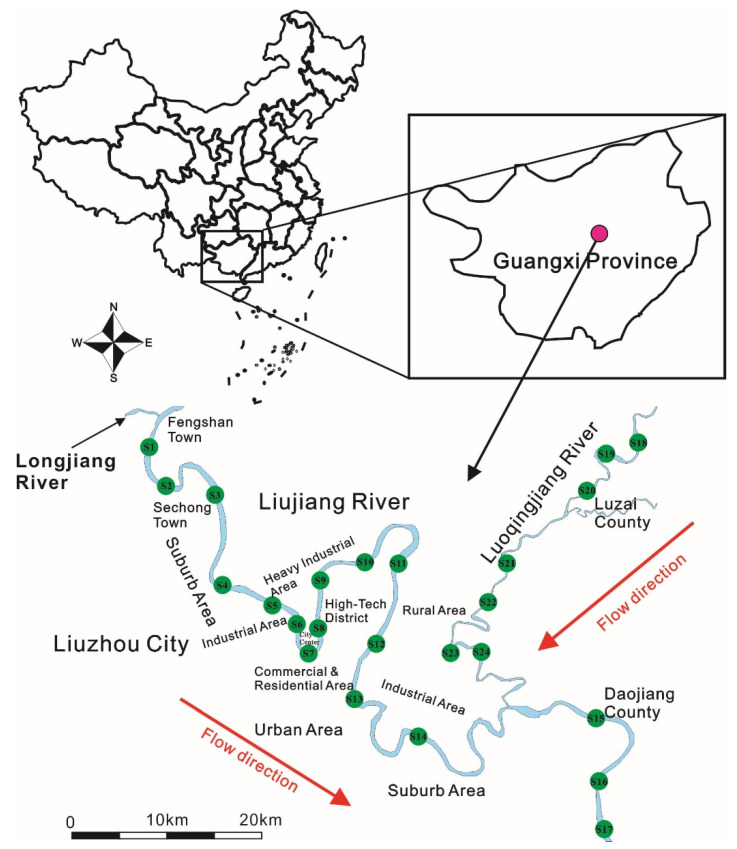
Sample plots in Liujiang River Basin [2].

The drastic environmental fluctuations, i.e., the variations in water level, water chemistry, and hydrodynamic conditions, were usually related to the input of rainfall, which may have the greatest impact on varying the bioavailability of metals in the sediments of rivers, so that the seasons that are divided by rainfall should be a primary consideration in this study, instead of the natural seasons. More than 80% precipitation of Liujiang River Basin was mainly centralized from March to June. During the continuous rainfall in these months, lots of sediment entered Liujiang River Basin and turned the surface water into yellow. The color of the surface water would return green once the continuous rainfall between March and June finished. As the indicator of the intensive rainfall between March and June, the color of the surface water should be also treated as an indicator to express the alternation of the wet and dry season. In order to heavily differentiate the environmental background in Liujiang River Basin, the sample collection was conducted right before and after, respectively, the continuous rainfall between March and June. The continuous rainfall in Liujiang River Basin gradually died down with the color of the surface water just returning green in the middle of May 2019, so that the sampling collection was conducted between 12 and 18 May 2019, which is conducive to maximizing the influence of continuous rainfall in the wet season. The sampling collection of the dry season was determined between 15 and 20 February 2020, which avoided the influences of the continuous rainfall and lots of sediments entering into Liujiang River Basin. Twenty-four sampling sites were selected along the Liujiang River and its tributary Luoqingjiang River, which covered the entire Liujiang River Basin (Figure 1). The sampling always kept away from the outlets of wastewater discharging to minimize the anthropogenic impacts and the surface sediments were sampled directly from the river bed (0–5 cm) by using a grab sampler. A sample was evenly collected and combined from three subsamplers in each plot within 100 m. A total of 24 test samples of surface sediment in the river bed were obtained from S1 to S24. The sediment samples were preserved in polythene self-sealing bags and transported at −20 °C after sampling. The physicochemical parameters of the aquatic environment, including pH, Eh (Redox potential), Dissolved Oxygen (DO), electrical conductivity (EC) and turbidity, were simultaneously determined with the multi-parameter water quality probe, which aims to explore the impacts of environmental fluctuation on metals’ bioavailability in sediments.

### 2.2. Sample Preparation and Analysis

The sediment samples were prepared with freeze-drying (−80 °C, 72 h), sieving (0.15 mm nylon mesh) and digesting (${\mathrm{HNO}}_{3}+\mathrm{HCl}+\mathrm{HF}$, 5:4:1 *v*/*v*, 140 °C and 6 h), respectively, for the total content testing, which are detailed in our previous studies [1,2].

The geochemical fractions of metals were extracted in accordance with the sequential extraction procedure (SEP) of Tessier, which divided metals into 5 forms, namely exchangeable (F1), carbonate-bound (F2), reducible (F3), oxidizable (F4), and residual fractions (F5). The detailed description of this SEP can be found in these studies [1,2]. To obtain the percent recovery of the metals using the adopted SEP, the total metals concentrations were compared with the total concentrations of the metals in the five SEP-derived fractions.

Cd, Cr, Cu, Pb, and Zn were analyzed by the inductively coupled plasma mass spectrometry method (ICP-MS, Thermo X series, ThermoFisher, Waltham, MA, USA), while Hg and As were measured with the atomic fluorescence spectrometry method (AFS-920, Titan Instruments, Shanghai, China). Organic matter was tested by the elemental analyzer (Vario EL-III, Elementar, Germany) and sediment particle size was tested by laser particle size analyzer (Mastersizer 2000, Malvern, USA).

All analyses were performed in triplicate and averaged as the final result.

### 2.3. Quality Assurance and Quality Control

The standard, blank, and parallel samples were rigorously incorporated into the samples testing to meet the procedures of quality assurance and quality control, which could also be found in our previous studies [1,2]. The recovery rates of total metals’ contents were reported between 95–105%, while the average recovery percentage of the total concentrations in the five fractions of metals compared with the total concentrations of metals was 93.25% ± 5.67%, which qualified for QA/QC compliance.

### 2.4. The Calculation of Metals Bioavailability

The exchangeable and carbonate-bound forms of metals were commonly considered to be highly bioavailable to aquatic biotas, so that they were widely used to calculate the bioavailability index of metals (*BI_M_*), which could be also employed in evaluating the potential risk and toxicity of heavy metals in sediments [3]. The *BI_M_* values of individual metals’ bioavailability index can be calculated using the following equation:

$$BI_{M}=\frac{C_{F1}+C_{F2}}{C_{ERL}}$$

where *C_F_*_1_ and *C_F_*_2_ represent the metals’ concentrations (mg/kg) of exchangeable form and carbonate-bound form, respectively. Moreover, *C_ERL_* is the threshold of metals’ concentration in sediments and was determined on the basis of the effects range-low (ERL) levels specified in the Sediment Quality Guidelines (SQGs) developed by the National Oceanic and Atmospheric Administration; specifically, Cu = 34 mg/kg; As = 8.2 mg/kg; Zn = 150 mg/kg; Cd = 1.2 mg/kg; Hg = 0.15 mg/kg; Cr = 81 mg/kg; and Pb = 46.7 mg/kg [29]. *BI_M_* can be used to evaluate the potential ecological risk and toxicity of metals in sediments, which when higher than 1 indicate higher bioavailability, potential risks, or toxicity; by contrast, values lower than 1 indicate lower bioavailability, potential risks, or toxicity.

### 2.5. Statistical Analysis

The correlation analysis was conducted to uncover the interactions between multiple environmental parameters, given the better applicability for the continuously environmental monitoring, Pearson’s correlation was adopted in this study. The correlations between metals’ speciations and bioavailability index (BI) were calculated with the help of SPSS 20. The histograms were output with Origin Pro 8 originally, while further processing with Coreldraw X4.

## 3. Results

### 3.1. The Properties in Sediments and Their Overlying Water

The properties of the overlying water and sediment are given in Table 1. For overlying water, the range of pH was 7.93–8.53 and 6.79–8.48, respectively, in the dry season and wet season, which suggested an overall leaning to alkalinity in the aquatic environment, regardless of the seasonal changes, while the slight elevation in mean pH in the dry season may be attributed to the more dominant recharge from the alkaline underground water in the karst area. The Eh and oxygen content of water are 144.74 mV and 10.84 mg/Lin the dry season and 114.03 mV and 7.2 in the wet season, respectively, both of which are significantly higher in the dry season. The mean values of EC reached 180 μs/cm in the dry season and 164 μs/cm in the wet season, respectively, which suggested a higher natural ions’ content in the dry season. For sediments, they were predominantly composed of silt, the main grain size of sediment were, respectively, 25.17 μm in the dry season and 21.26 μm in the wet season. The elevation of grain size in the dry season should be closely related to the dramatic loss of clay, the proportions of which were even approaching zero in most plots. The concentrations of organic matters were generally low in Liujiang River Basin, particularly in the dry season, which were below 0.7%.

### 3.2. The Distribution and Speciations of Metals in Sediments

**Figure 2:**
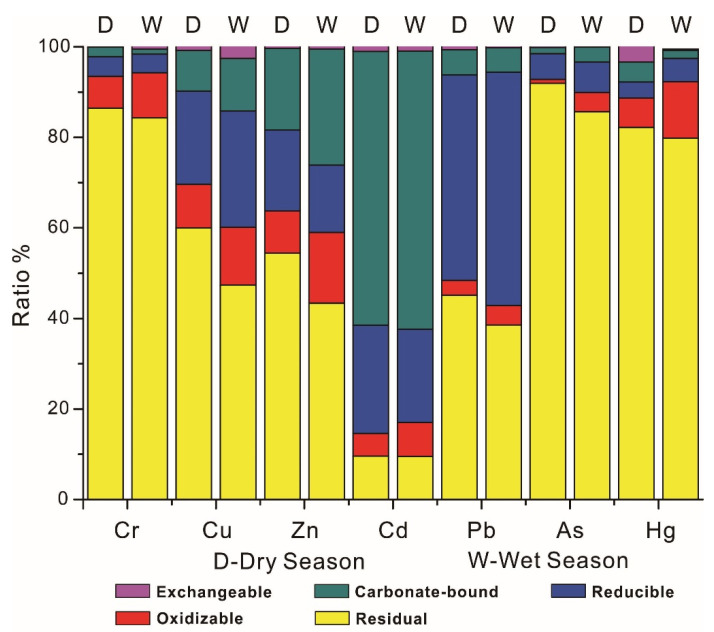
The speciations of metals in sediments of Liujiang River Basin.

The distribution and speciations of heavy metals in sediments are shown in Table 2, Figure 2 and Figures S1 and S2. Almost all metals were found to be generally higher in the wet season, but they shared the same decreasing order (Zn > Cr > Pb > Cu > As > Cd > Hg) in different seasons, which suggested an identical type but varying degrees of pollution with the changing seasons. Compared with the sediment quality baseline [30,31], Cu and Pb were mostly lower than the TEL (threshold effect level), while other metals were generally between the TEL and PEL (probable effect level), indicating a low risk of the metals exerting negative effects on the benthos. Zn and Cd were significantly higher than their corresponding background values, while other metals were close to or lower than their corresponding background values. Therefore, the concentration of Cd and Zn in the sediments of Liujiang River Basin may be elevated by human activities. For the wet season, metals were commonly decreased from the upstream to downstream of the Liujiang River, their higher concentrations were mainly centralized in the upstream of Liujiang River (S1–S2), which may be tightly related to the rapid accumulation of contaminated sediments that move down from the Longjiang River in the wet season. For the dry season, the concentrations of metals in the upstream were significantly decreased, which means that this area no longer had a higher concentration of metals, while the area with a higher concentration of metals was determined to be the midstream of Liujiang River (S7–S14), which may be a result of from the transfer of contaminated sediments after the wet season. In general, the concentrations of metals in Liujiang River were commonly lower than that in Luoqingjiang River, regardless of the alternation of seasons, which suggested a higher environmental quality in Luoqingjiang River.

The speciations of metals in sediments are shown in Figure 2 and Figures S1 and S2 and Tables S1 and S2. Apart from Pb and Cd, metals were mostly in the residual form. More than 80% of Cr, As and Hg were found in residual form, which indicated the dominance of natural sources. Cu and Zn were determined to be a little lower in proportion in the residual form, as both of them were around 50%, manifesting a certain amount of exogenous input. The residual form of Pb and Cd were determined to be not their dominant form, their dominant forms were the reducible and carbonate-bound form, respectively, which suggested an intense influence of human activities. The residual forms of all metals were found to be elevated, with their total concentration shrinking from the wet season to the dry season, which suggested the exogenous metals were prone to losing their non-residual form after the wet season. Therefore, there is reason to believe that the purification of contaminated sediments in the karstic river might mainly occur in the dry season, instead of the wet season. For the non-residual form, the oxidizable form of all metals were shrinking as their total concentration in the dry season, which highlighted the oxidizable form should be treated as the predominant form of exogenous metals’ loss. Despite the fact that concentrations of all metals in reducible form were still found to be decreased in the dry season, the proportion of metals in this form were not entirely reduced in the dry season, such as Cr, Zn and Cd, the ratio of which were elevated in the dry season. The carbonate-bound forms were mostly reduced in the dry season, but the significant elevation of Cr and Hg could still be found in the dry season. For exchangeable form, most metals were found to be increased in the dry season, among them, the content of As, Pb and Hg in exchangeable form was determined to be three, four and ten times as high as that in the wet season, respectively. The significant elevation of metals in these two forms in the dry season suggested that the purification of contaminated sediments after the wet season should not directly result from the loss of exogenous metals from non-residual form, but may be more related to the destabilization of metals’ transformation, while the carbonate-bound forms and exchangeable form should be treated as the critical mediation, especially the exchangeable form, which could accommodate the destabilized metals during the non-residual form shrinking.

### 3.3. The Bioavailability of Metals in Sediments

**Figure 3:**
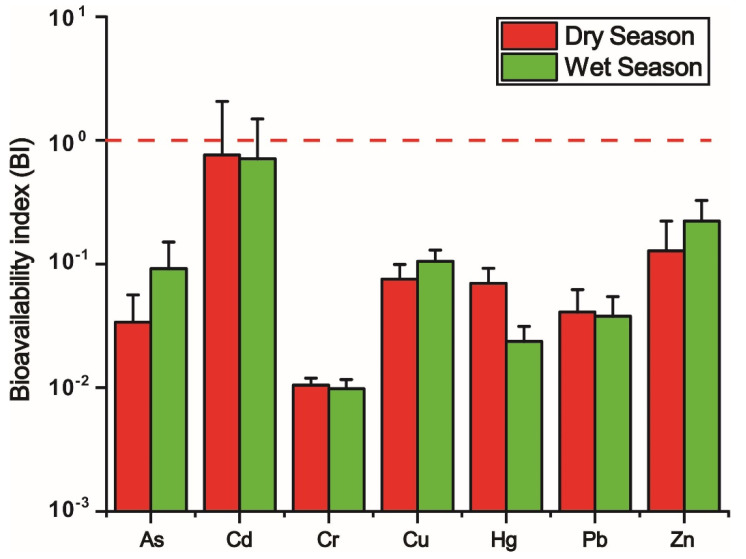
Bioavailability index (BI) of metals in sediments.

The BI of metals in sediments are shown in Figure 3. For the BI values, the decreasing orders in the dry season and wet season are as follows: Cd > Zn > Cu > Hg > Pb > As > Cr and Cd > Zn > Cu > As > Pb > Hg > Cr, respectively, the variation of which highlights the conspicuous seasonal changing of the bioavailability of Hg and As. The BI values of Cd are confirmed to be the highest, regardless of the seasons, which indicate their superiority of bioavailability in the Liujiang River Basin. For the mean of BI values, they are all less than the threshold of 1, regardless of the species of metals and the seasons, which suggest the bioavailability of metals is not high in the whole basin. However, it is still worth noting that there are still some BI values of Cd found to be extremely high, the maximum of which even reaches six in the dry season and four in the wet season, respectively. All of this suggests that the high risk of Cd contamination should be confined to a small scale. Therefore, reducing the bioavailability of Cd on a small scale, instead of the whole basin, should be the priority to remediate the Cd contamination in the Liujiang River Basin. Given the close relationship between metals’ bioavailability and environmental security, there must be vigilance to the elevation of the bioavailability of individual metals in the dry season.

## 4. Discussion

### 4.1. Basis for the Seasonal Variations of Metals Bioavailability

Exchangeable and carbonate-bound forms were considered to be the highly bioavailable forms of metals, which has a vital impacts on the geochemical cycles of metals [3]. Despite they were the critical forms to impact metals’ bioavailability, both of them could not determine the variation of metals’ bioavailability on their own [2], so that it is of great value to clarify their interactions with metals’ bioavailability. For carbonate-bound forms, although their concentrations were mostly reducing in the dry season, their correlations were confirmed to be significant among all metals either in the wet and dry season, the correlation coefficients of which were significantly higher than that of other metals forms (Table 3). All of this suggested this form could offer the consistently, stably and widely supports to metals’ bioavailability. For each metal, only the carbonate-bound form of Cr and Hg was found to be elevated Tables S1 and S2 (Supplementary Materials); particularly for Cr, its elevation of the carbonate-bound form further highlighted the predomination of the carbonate-bound form on its bioavailability, which was suggested by its higher correlation coefficient in the dry season, while the lower correlation coefficient in the dry season suggested the elevation of Hg in carbonate-bound form failed to aggravate the impact of the carbonate-bound form on its bioavailability, which mainly resulted from the sharp rise of Hg in an exchangeable form that impaired the predomination of carbonate-bound form on its bioavailability. With the exception of Cr and Hg, the carbonated forms of other metals were all decreased, which, however, did not significantly reduce their correlation coefficients; particularly for Cu, its correlation coefficients were even elevated, which highlighted the intense dominance of the carbonate-bound form on manipulating metals’ bioavailability throughout the year.

For exchangeable forms, despite the fact that they were considered to be more bioavailable to aquatic biotas, their support to metals’ bioavailability could often not be compared with other forms, which was suggested by their lower correlation coefficients, particularly, the correlation coefficients of some metals in exchangeable forms were even found to be lower than that in reducible forms and oxidizable forms (shown in Table 3), which mainly resulted from the overall lower concentrations of metals in exchangeable forms. The elevation of exchangeable forms in the dry season was confirmed to be Pb, Cd, As, and Hg (Tables S1 and S2), but their rising of correlation coefficients was only determined to be Pb and Cd. Although the correlation coefficient of Hg in exchangeable form failed to increase with its content rising in the dry season, the reversion of its correlation coefficient makes the exchangeable form of Hg become the dominant form on manipulating its bioavailability, which suggested the increasing of metals in exchangeable form should be actually beneficial to upgrade their impacts on manipulating metals’ bioavailability, but their significance should be tightly related to their elevation, which was well expressed on As. The elevated ratio of As in exchangeable form was found to be significantly lower than that of Pb, Cd, and Hg in the dry season (Tables S1 and S2), so that the elevation of its correlation coefficient was confirmed not to have appeared in the dry season, which suggested the low ratio rising of exchangeable form may be hard to significantly promote their impacts on metals’ bioavailability. For Cr, Cu, and Zn, their exchangeable forms were determined to be shrinking in the dry season (Tables S1 and S2), among them, the reduction in Cr and Cu in exchangeable form significantly degraded their impacts on metals’ bioavailability, which made their correlations rapidly reduce to the state of non-significance with BI in the dry season. However, for Zn, its decreased ratio of exchangeable form was relatively lower than other metals, so that the downregulating of its correlation coefficients did not appear in the dry season, which suggested the shrinking of metals in exchangeable forms could substantially impair their influences on metals’ bioavailability, but their significance were also tightly related to their decreased ratio. On the whole, the large fluctuations of exchangeable forms should be critical to vary the impacts of exchangeable forms on metals’ bioavailability.

In general, the variations in metals’ bioavailability after the wet season were mainly determined by the predominance of metals in exchangeable and carbonate-bound form on their bioavailability, which were well expressed on almost all metals. For Pb, Cu, Zn and Cr, the higher correlation coefficients highlighted the predominance of carbonate-bound form in the dry season, so that the fluctuation of their content in carbonate-bound form determined the trend of their bioavailability after the wet season. Among them, the elevation of Pb and Cr bioavailability in the dry season should be tightly connected to their increased content in carbonate-bound forms, while the reduction in Zn and Cu bioavailability in the dry season should largely result from their content shrinking in carbonate-bound forms. For Hg, the higher correlation coefficient indicated the dominant form should be its exchangeable form in the dry season, so that its bioavailability rising in the dry season should be attributed to its elevation of exchangeable form. For As, although its exchangeable form in the dry season has significantly increased three times higher as that in the wet season, its bioavailability was not found to be elevated in the dry season. The most critical reason for this should be considered to be that the exchangeable form was not the dominant form for its bioavailability, which also confirmed by its significantly lower correlation coefficient than that of other forms in the dry season. Therefore, it could be concluded that the escalation in exchangeable form could also make it hard to exert their impacts on varying their bioavailability, once the predominance of exchangeable form on metals’ bioavailability was determined to be deficient. It definitely did not mean to negate the possibility that the exchangeable form may independently vary metals’ bioavailability, the independence of which, on the contrary, could be found on Cd. For Cd, despite the higher correlation coefficient manifested, its dominant form was carbonate-bound form, its variation of bioavailability could be hard to obtain supports from the slight seasonal variation in the carbonate-bound form; particularly, its variation was determined to be reduced after the wet season. Therefore, there is a reason to believe that the elevated bioavailability of Cd in the dry season should result from the aggregation of exchangeable form after the wet season. In fact, although the exchangeable form was not the dominant form for Cd bioavailability, its aggregation in the dry season significantly aggravated its regulation on varying Cd bioavailability, which was confirmed by its elevated correlation coefficient after the wet season. With the background that the manipulation of carbonate-bound form on Cd bioavailability remains unchanged in different seasons, the aggregation of the exchangeable form became a critical motivation to uphold the elevation of Cd bioavailability after the wet season. Therefore, the independent manipulation of exchangeable form on metals’ bioavailability should be more likely to stand out on the background that the manipulation of the dominant metals’ form remains constant.

### 4.2. The Destabilization of Metals in Strongly Bound Form

Metals in reducible and oxidizable forms were generally stable and relatively non-bioavailable, so it could be very hard for them to exert their impacts on threatening environmental security directly. However, once the destabilization relevant to the reversed transformation of metals take place with the influence of environmental fluctuations [26], the aggregation of metals in exchangeable form and carbonate-bound form, which resulted from the destabilization of reducible and oxidizable forms, would have an indirect impact on metals’ bioconversion. In view of the great differences of metals’ transformation, the reversed transformation of metals in reducible and oxidizable forms should be detailed to clarify their interactions with metals’ bioavailability (Table 3). For oxidizable forms, except for Cu, the correlations of BI were found to be significant with almost all metals in the wet season, while the correlations of BI were confirmed to be only significant with Cd, Hg, Pb and Zn in the dry season; additionally, among them, the vanished significant correlations were only connected to As and Cr in the dry season. Based on previous studies, metals in oxidizable form were not entirely bound in strong site, but about 1–10% of them still belonged to weakly bound sites [32,33], which shared the higher possibility of loss in environmental fluctuations, so that these weakly bound metals would lose preferentially under the background of the destabilization of oxidizable forms. However, the loss of these weakly bound metals would inevitably enhance the stability of metals in oxidizable form, and then impair their possibility of destabilization, which eventually resulted in the degradation of the impacts of the oxidizable form on metals’ bioavailability. Compared with other metals, the correlations of As and Cr in oxidizable forms were confirmed to be weaker in the wet season, which indicated their ability to influent their bioavailability by means of the destabilized oxidizable form should be inherently weak. Additionally, all of them further experienced their loss in weakly bound sites of oxidizable form after the wet season (Tables S1 and S2), and then largely reduced their destabilization from oxidizable form, so that the impacts of their oxidizable forms on their bioavailability were greatly degraded, which was directly expressed in the extinction of their significant correlations of oxidizable form. Therefore, there is a reason to believe that the loss of metals in oxidizable form after the wet season should be responsible for the degradation of their influences on metals’ bioavailability.

Although the impacts of metals in specific form on metals’ bioavailability were confirmed to be closely related to their content in this form, it did not mean that metals’ bioavailability could be only varied by their content in a specific phase. Given the impacts of environmental variations on the stability of metals in different forms, environmental variations should also play an important role in varying the impacts of metals’ speciations on metals’ bioavailability, the concept of which was formerly proposed [1]. In this study, the correlation coefficients of Cd, Pb and Zn in oxidizable form were not found to be diminished by their content shrinking in oxidizable form after the wet season, all of their correlation coefficients were still significantly higher in the dry season, which suggested that the shrinking of oxidizable form in the dry season did not have a significant impact on their stability, but this should be related to the enhanced interactions between metals in oxidizable form and their bioavailability that resulted from the decomposition of organic matters in the dry season. The oxidization degree was generally higher in the dry season, which confirmed by their higher DO and Eh in the aquatic environment. Metals in oxidizable form predominantly result from metals combining with organic matter [2], so that the reduced stability of organic matter in oxidizing conditions would aggravate the destabilization of metals in oxidizable form, which would also inevitably elevate their reversed transformation to bioavailable parts, and then upgrade their support of metals’ bioavailability. Therefore, the seasonal fluctuations of Eh and DO should play a critical role in enlarging the impacts of metals in oxidizable phases on metals’ bioavailability.

For reducible forms, their significant correlations were found to be As, Cr, Cd, Pb and Zn in the wet season, As and Pb in the dry season, respectively, which suggested that the loss of metals in reducible forms significantly degraded their impact on metals’ bioavailability. Different from metals in oxidizable form, the extinction of significant correlations were confirmed to be Cd, Cr and Zn in the dry season, but their correlation coefficients were higher than other metals in the wet season, which suggested the impacts that resulted from the shrinking of reducible phases on metals’ bioavailability should not be directly related to their own ability to destabilize. In fact, the impacts of reducible forms on metals’ bioavailability were totally dependent on their undifferentiated destabilization to bioavailable parts in accordance with their generally higher non-bioavailability, so that the high rate of loss of metals in reducible forms should be the direct embodiment of their support for metals’ bioavailability. Although the reducible form was determined to be overall shrunk in content after the wet season, the increased ratios of Cd, Cr and Zn still suggested their rate of loss that resulted from their destabilization could not compare with that of other metals (Tables S1 and S2). The relatively lower outputs that destabilized from Cd, Cr and Zn in reducible phases directly restricted their impacts on their bioavailability, particularly in the dry season, which became the critical reason that their correlations were not found to be significant in the dry season any more. Metals in reducible forms are predominantly combined with iron and manganese oxides [33], which are relatively stable in oxidizing condition, so that metals in reducible forms should be more likely to destabilize to bioavailable parts in the wet season with lower Eh and DO, instead of the dry season, which just corresponded to the higher correlation coefficients with BI in wet season. The decreased correlation coefficients of metals in reducible phase after the wet season indicated the impacts of metals in reducible form on metals’ bioavailability should be completely dominated by their content in this form.

Despite the fact that metals in residual form were non-bioavailable, they could still affect metals’ bioavailability by weathering the primary minerals, which were also generally expressed on their significant correlations with BI. For As, Hg and Zn in residual form, their correlation coefficients were even found to be higher than that of other speciations, which suggested that the impact of the residual form on metals’ bioavailability should be not overlooked. Given that microbes commonly participated in the processes of minerals’ weathering [2], the support of metals in residual form on metals’ bioavailability should be closely related to the efficiency of microbial mineralization. The activity of microorganisms was widely confirmed to be higher in the wet season [19,23], which mainly resulted from the suitability of temperature for microorganisms growing in this season, so that the impacts of residual form on metals’ bioavailability should be aggravated only in the wet season. However, the correlation coefficients between metals in residual form and BI were found to be increased after the wet season, instead of decreased, which suggested the suitability of temperature should be not the dominant factor in manipulating the efficiency of microbial mineralization. In fact, temperature is not the only factor that regulates the efficiency of microbial mineralization. On the contrary, the centralized substrates could be also treated as the main driving force to improve the mineralization efficiency [2]. Although the suitability of temperature in the dry season could not compare with that in the wet season, their microbial mineralization would not stop either. The centralization of metals in residual form, which resulted from the loss of exogenous metals in non-residual forms, would also play an important role in enhancing their efficiency of mineralization, and then aggravating their impacts on metals’ bioavailability in the dry season. However, once the centralization of metals in residual form was too low in the dry season, their slight elevation would not be able to compensate for the decreased activity of microorganisms on the efficiency of mineralization, and then to make no contribution to increase their impacts on metals’ bioavailability in the dry season, which was well-expressed in the vanished correlation of Cr in the dry season. Therefore, a conclusion could be reached that the support of metals in residual form on metals’ bioavailability should be strictly restricted by their centralization in the dry season.

Generally, the stability of iron and manganese oxides was low in the wet season with the effect of the low Eh and DO, which have the possibility of destabilization, so that the destabilization of metals in reducible form should be treated as a critical approach to manipulate metals’ bioavailability, the predominance of which was confirmed by their higher correlation coefficient than that of oxidizable form. For the dry season, the elevation of DO and Eh degraded the stability of organic matters, so that the destabilization of metals in oxidizable form became the vital approach to manipulate metals’ bioavailability, which was determined by their higher correlation coefficient rather than that of reducible form. With the exception of Cr, the upgrading of metals’ bioavailability in the dry season were all significantly supported by their oxidizable form (significant correlations with BI), which manifested the destabilization of oxidizable form played a dominant role on varying metals’ bioavailability. However, the predomination of the oxidizable form in regulating metals’ bioavailability was not invariable, but could be also reversed by their content shrinking, the best example of which is provided by Zn. The loss of Zn was determined to be intense in entirety after the wet season, which significantly degrades the possibility for the upregulation of its bioavailability. Despite the significant correlation confirmed by the support of oxidizable form on Zn bioavailability, it would be very hard to reverse the impacts of the overall shrinking on its bioavailability by only relying on the destabilization of its oxidizable form, which ultimately resulted in the reduction in Zn bioavailability with its content shrinking in the dry season. Therefore, the impacts of the oxidizable form on metals’ bioavailability should be more likely to be confined to the metals that are less involved in the seasonal shrinking. For Cr, the non-significant correlation suggested its bioavailability had not received the support from its oxidizable form, so that the elevation of its bioavailability should be blamed on the aggregation of exogenous Cr. In fact, the emission of exogenous Cr was reported to be widespread in Liujiang River Basin [25,28], but it preferred to stay in the water column, instead of sediments [26], which resulted in the relatively low ratio of Cr in non-residual forms. However, with the enhanced recharge from the alkaline groundwater in the karst catchment after the wet season, the elevation of carbonate in the water column aggravated the combination of exogenous Cr with carbonate ions, so that the carbonate-bound Cr in the water column would gradually aggregate to sediments in light of their low solubility, which just corresponded to the significant increase in Cr only in carbonate-bound form. Therefore, the elevation of Cr bioavailability in the dry season should be largely related to the aggregation of exogenous Cr.

### 4.3. The Regulation of Seasonal Variations on Metals Bioavailability

The discharge of contaminated sediments was commonly considered to be a huge threat to the environmental security of watersheds. The gradually strengthened runoff moved large amounts of contaminated sediments, which were left over in Longjiang River after the previous incident of Cd pollution in 2012, to the watershed of Liujiang River in the wet season. With the increase in the flow area in the confluences between Longjiang river and Liujiang River, the transporting capacity of the river decreased with the flow rate declining, so that the pour deposition of the polluted sediment from Longjiang River came out primarily in the upstream of Liujiang River, which also shaped the decreasing trends of most metals, particularly Cd, from upstream to downstream in the watershed of Liujiang River. Given the large exogenous metals in contaminated sediments, the deposition of contaminated sediments would have significantly elevated the ratio of metals in non-residual forms, and then aggravated their impacts on metals’ bioavailability, which also explained that metals in non-residual forms could manipulate the bioavailability of most metals in the wet season. However, the gradual end of the runoff back to normal in the dry season with the end of the wet season directly terminated the discharge of contaminate sediments from the upstream of the watershed of Liujiang River. In the absence of contaminated sediment replenishment, the contaminated sediments that were previously deposited would lose gradually with the effect of turbulent flow in river, but the loss of sediments was not even, the lightweight fine grains of sediments would generally obtain the priority. As the finest part in sediment, the reduction in clay sediments naturally became the manipulator on reshaping metal contamination of river after the wet season. Given the more intense absorption for exogenous metals from fine grains in sediments [1,33], instead of their coarse grains, the loss of clay sediments would aggravate the loss of metals in non-residual forms, and then eventually turn the composition of metals’ speciations into the residual form with higher stability, which was confirmed by the greater centralization of residual forms in the dry season. However, the coarsened sediment would not only impair the aggregation of exogenous metals, but also decrease the content of multiple active substances that with the affinity of exogenous metals, especially organic matters [1], were confirmed by the reduction in organic matters in sediments after the wet season. Based on previous studies, the critical stabilization of exogenous metals in sediments were predominantly offered by organic matters [34], so that the decrease in organic matters that resulted from sediments’ coarsening would impose the impacts on the destabilization of exogenous metals in sediments, particularly under the overall elevation of dissolved oxygen in river, the destabilization of organic matters would definitely aggravate the destabilization of metals, which also explained that metals in oxidizable form offered more support for metals’ bioavailability in the dry season. Without the stabilization of sufficient organic matters, the destabilization of metals should be more likely to appear in the form of exchangeable and carbonate-bound, which also became the critical mechanism whereby the bioavailability of most metals was only elevated in the dry season.

The smart water conservancy was proposed recently to manage metals’ contamination in the watershed via the approach that drove the transportation of contaminated sediment [35]. Despite the fact that the regulation of water conservancy may work in avoiding the excessive aggregation of contaminated sediments, the coarsening sediments after the regulation of water conservancy may also aggravate the destabilization of metals in sediments, and then increase metals’ bioavailability in sediments, which should be treated as a potential threat in the environmental security of Liujiang River Basin. In addition, metals’ bioavailability in sediments was susceptible to the elevation of Eh and DO in river [26], so that the fluctuation of water chemistry should be not overlooked during the regulation of metals’ contamination in the watershed of Liujiang River. The monitoring of water chemistry and metals’ bioavailability should be simultaneously incorporated into the regulation of metals’ contamination in the watershed of Liujiang River, which is of great value to improve the regulation of metals’ contamination in river and safeguard the environmental security.

## 5. Conclusions

Metals’ concentration in sediments were overall high in the wet season, but low in the dry season. The significantly reduced ratio of metals in non-residual forms was largely related to the overall reduction in metals in oxidizable and reducible forms after the wet season. However, the elevated BI indexes of most metals suggested their increased bioavailability in the dry season, which should be closely related to their corresponding elevations in carbonate-bound and exchangeable forms after the wet season. The variations in metals’ bioavailability were primarily related to their predominance in exchangeable and carbonate-bound form. The higher correlation coefficients suggested the destabilization of the oxidizable form should be treated as a critical approach to the impacts on metals’ bioavailability after the wet season. In view of the fact that sediments’ coarsening would impact on the destabilization of exogenous metals in sediments, the reduction in clay sediments should be responsible for the elevation of metals’ bioavailability after the wet season. Therefore, the monitoring of metals’ bioavailability in sediments should be indispensable to prevent metal contamination from increasing its threat on the aquatic environment of the river, especially after the wet season.

## Figures and Tables

**Table 1:** The properties of sediments and overlying water. DO: dissolved oxygen; EC: electrical conductivity; Eh: redox potential; OM: organic matter; Mz: mean grain size.

**Overlying Water**	**DO**	**EC**	**pH**	**Eh**	**Turbidity**
	**mg/L**	**μs/cm**		**mV**	**NTU**
Dry Season	10.16–11.82	147.9–227.0	7.93–8.53	114.4–179.2	1.57–11.7
	10.84	180.65	8.13	144.74	4.27
Wet Season	6.22–8.43	141.5–199.7	6.79–8.48	94.89–161.1	8.13–28.1
	7.20	164.32	7.81	114.03	13.61
**Sediment**	**OM**	**Mz**	**Clay**	**Silt**	**Sand**
	**%**	**μm**	**<4 μm%**	**4–63 μm%**	**>63 μm%**
Dry Season	0.38–1.37	7.92–32.95	0.02–35.77	64.23–96.96	0–8.43
	0.67	25.17	2.92	92.57	4.51
Wet Season	0.37–1.37	10.24–27.63	15.74–27.63	62.74–79.84	0.05–7.24
	0.72	21.26	22.16	74.05	3.79

**Table 2:** Heavy metal concentrations in sediments in Liujiang River Basin. BSG: background values of soil in Guangxi, China; TEL: threshold effect level; PEL: probable effect level.

Location		Cd	Pb	Cr	Cu	Zn	As	Hg
mg/kg								
**BSG**		0.267	24	82.1	27.8	75.6	20.5	0.152
**Wet season**	Min–Max	0.44–6.36	17.74–43.31	27.64–91.00	19.98–35.86	68.47–196.96	8.29–69.76	0.09–1.32
	Mean	1.27	30.10	53.53	25.20	124.93	23.24	0.19
**Dry Season**	Min–Max	0.45–8.74	21.28–55.73	29.86–59.98	20.19–37.67	56.56–208.99	10.00–28.18	0.09–0.47
	Mean	1.27	30.10	39.70	26.46	96.71	17.79	0.15
**TEL**		0.6 ^a^	35 ^a^	42 ^a^	36 ^a^	123 ^a^	7.2 ^b^	0.17 ^a^
**PEL**		3.5 ^a^	91 ^a^	160 ^a^	197 ^a^	315 ^a^	42 ^b^	0.49 ^a^

^a^ The freshwater sediment quality of Canada; ^b^ The sediment quality criteria of Hong Kong.

**Table 3:** The correlations between the bioavailability and speciations of metals in dry season and wet season.

**Dry Season**	**As**	**Cd**	**Cr**	**Cu**	**Hg**	**Pb**	**Zn**
	**BI**						
**F1**	**0.673** **	**0.922** **	**0.098**	**0.123**	**0.692** **	**0.526** **	**0.644** **
**F2**	**0.997** **	**1.000** **	**0.997** **	**0.994** **	**0.659** **	**0.992** **	**1.000** **
**F3**	**0.720** **	0.21	−0.08	0.149	0.020	**0.689** **	0.272
**F4**	0.093	**0.982** **	0.318	0.131	**0.624** **	**0.785** **	**0.902** **
**F5**	**0.730** **	0.38	0.300	0.108	**0.674** **	**0.487** *	**0.742** **
**Wet Season**	**As**	**Cd**	**Cr**	**Cu**	**Hg**	**Pb**	**Zn**
	**BI**						
**F1**	**0.784** **	**0.689** **	**0.842** **	0.389	**0.840** **	0.137	**0.476** *
**F2**	**1.000** **	**1.000** **	**0.820** **	**0.930** **	**0.919** **	**0.998** **	**1.000** **
**F3**	**0.763** **	**0.977** **	**0.741** **	0.304	0.258	**0.836** **	**0.896** **
**F4**	**0.468** *	**0.856** **	**0.656** **	0.173	**0.701** **	**0.727** **	**0.737** **
**F5**	**0.621** **	0.372	**0.615** **	0.389	**0.414** *	0.236	**0.674** **

* Correlation is significant at the 0.05 level (2-tailed). ** Correlation is significant at the 0.01 level (2-tailed).

## Supplementary Materials

The following supporting information can be downloaded at: https://www.mdpi.com/article/10.3390/ijerph192214988/s1, Figure S1: The spatial distribution of metals speciations in dry season; Figure S2: The spatial distribution of metals speciations in wet season; Table S1: The metals speciations of Liujiang River Basin in dry season; Table S2: The metals speciations of Liujiang River Basin in wet season.
