# Supplementary material for: The Influence of the Reduction in Clay Sediments in the Level of Metals Bioavailability—An Investigation in Liujiang River Basin after Wet Season
Source: Int J Environ Res Public Health. 2022 Nov 14;19(22):14988. doi: 10.3390/ijerph192214988 (PMC9690423; doi:10.3390/ijerph192214988)
Supplement: Supplementary file 1 [file ijerph-19-14988-s001.zip › ijerph-1966686-supplementary.pdf]

The Influence of the Reduction in Clay Sediments in the Level of Metals Bioavailability—An Investigation in Liujiang River Basin after Wet Season

Xiongyi Miao [1,2,3], Jianping Liang [4], Yupei Hao [3,5,*], Wanjun Zhang [3], Yincai Xie [3] and Hucai Zhang [5,*]

[1] School of Geography and Environmental Science, Guizhou Normal University, Guiyang 550001, China
[2] School of Karst Science, Guizhou Normal University, Guiyang 550001, China
[3] Key Laboratory of Karst Dynamics, MNR&GZAR, Institute of Karst Geology, CAGS, Guilin 541004, China
[4] Guilin Meteorological Bureau of Guangxi, Guilin 541000, China
[5] Institute for Ecological Research and Pollution Control of Plateau Lakes, School of Ecology and Environmental Science, Yunnan University, Kunming 650500, China
* Correspondence: yphao66@126.com (Y.H.); zhanghc@ynu.edu.cn (H.Z.)

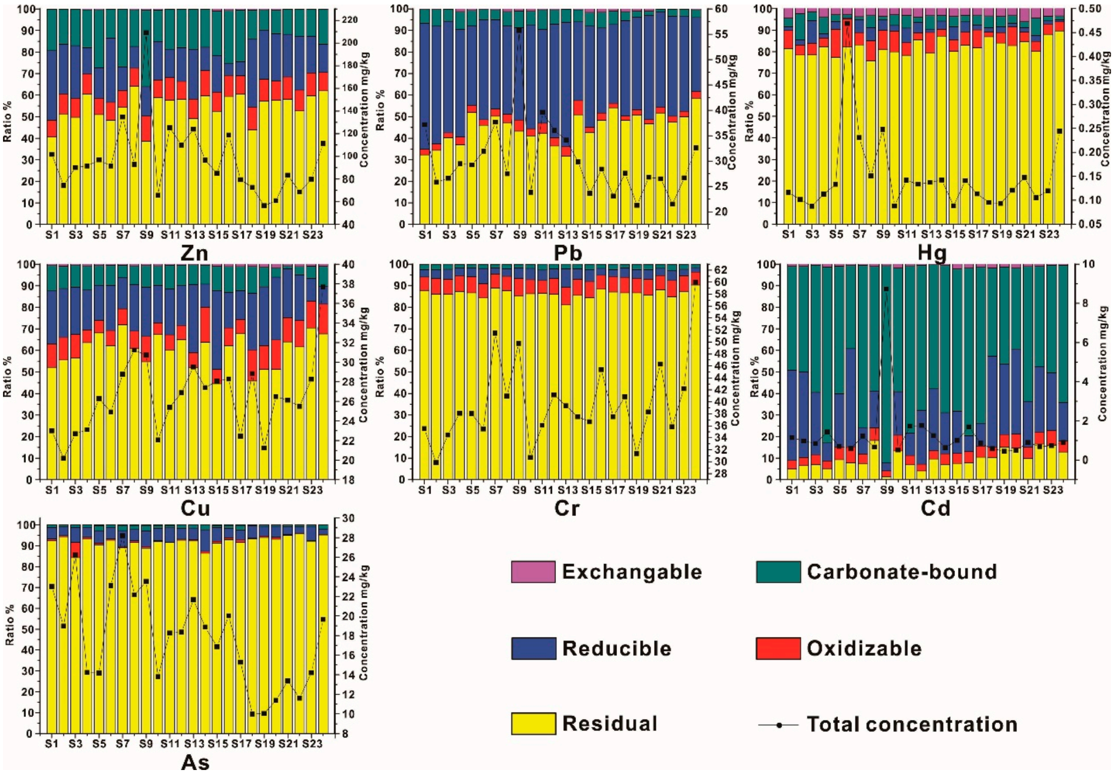

Figure S1. The spatial distribution of metals speciations in dry season.

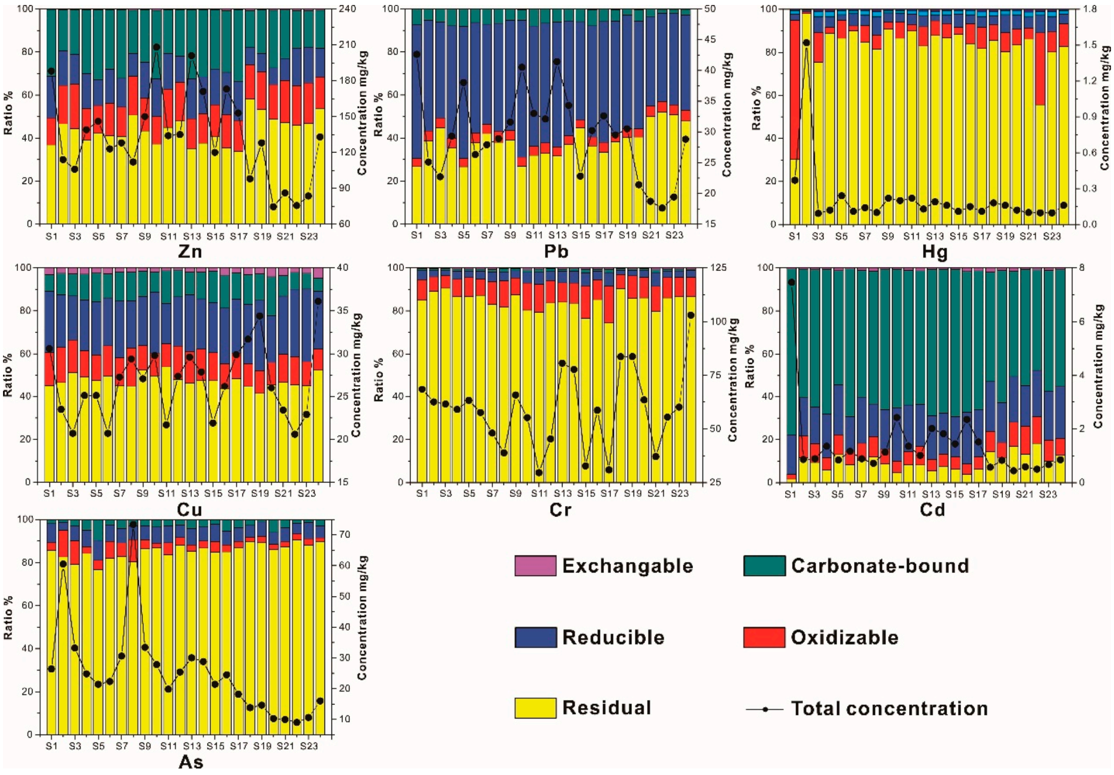

Figure S2. The spatial distribution of metals speciations in wet season.

Table S1. The metals speciations of Liujiang River Basin in dry season.

Metal	Exchangeable Fraction (mg/kg)		Carbonate-bound Fraction (mg/kg)		Reducible Fraction (mg/kg)		Oxidizable Fraction (mg/kg)		Residual Fraction (mg/kg)	
	Mean±SD	Ratio(%)	Mean±SD	Ratio(%)	Mean±SD	Ratio(%)	Mean±SD	Ratio(%)	Mean±SD	Ratio(%)
**Cr**	0.02±0.01	0.04	0.83±0.12	2.13	1.68±0.5	4.32	2.78±0.48	7.02	34.4±6.5	86.48
**Cu**	0.21±0.09	0.78	2.36±0.79	8.95	5.4±1.92	20.62	2.57±1.05	9.60	15.92±3.32	60.05
**Zn**	0.31±0.26	0.33	18.91±13.91	18.03	16.57±7.13	17.93	9.07±3.81	9.31	51.85±14.23	54.41
**Cd**	0.01±0.01	1.02	0.9±1.55	60.48	0.22±0.11	23.89	0.05±0.04	5.02	0.09±0.03	9.58
**Pb**	0.19±0.13	0.67	1.72±0.92	5.50	13.7±4.01	45.43	1.02±0.57	3.26	13.47±3.5	45.15
**As**	0.03±0.02	0.14	0.25±0.17	1.32	1.04±0.51	5.71	0.18±0.38	0.90	16.28±4.4	91.93
**Hg**	0.005±0.003	3.37	0.006±0.002	4.37	0.005±0.004	3.52	0.011±0.012	6.52	0.122±0.069	82.22

Table S2. The metals speciations of Liujiang River Basin in wet season.

Metal	Exchangeable Fraction (mg/kg)		Carbonate-bound Fraction (mg/kg)		Reducible Fraction (mg/kg)		Oxidizable Fraction (mg/kg)		Residual Fraction (mg/kg)	
	Mean±SD	Ratio(%)	Mean±SD	Ratio(%)	Mean±SD	Ratio(%)	Mean±SD	Ratio(%)	Mean±SD	Ratio(%)
**Cr**	0.28±0.09	0.53	0.53±0.09	1.04	2.12±0.68	4.12	5.14±1.16	9.98	45.64±13.53	84.32
**Cu**	0.64±0.29	2.53	2.91±0.78	11.63	6.5±1.51	25.68	3.21±0.64	12.79	11.91±1.86	47.36
**Zn**	0.57±0.27	0.47	33.91±16.32	25.64	19.63±9.4	14.85	19.16±4.31	15.66	53.05±11.84	43.38
**Cd**	0.01±0.01	0.95	0.95±1.13	61.43	0.29±0.29	20.57	0.08±0.02	7.51	0.09±0.02	9.54
**Pb**	0.05±0.04	0.15	1.72±0.79	5.45	16.15±6.14	51.53	1.3±0.31	4.3	11.18±1.34	38.57
**As**	0.01±0	0.08	0.72±0.46	3.25	1.52±0.8	6.73	1.22±1.64	4.3	19.22±10.24	85.64
**Hg**	0.0012±0.0005	0.34	0.0024±0.0008	1.73	0.0068±0.0018	5.14	0.02±0.05	12.47	0.15±0.22	79.85
